# Spontaneous resolution of a giant bulla in a patient presenting with COVID‐19 with presumed superadded bacterial infection

**DOI:** 10.1002/rcr2.70074

**Published:** 2024-12-03

**Authors:** Ian Y. H. Chan, Keir E. J. Philip, Thomas Tsitsias, Carl Reynolds

**Affiliations:** ^1^ NHLI Imperial College London London UK

**Keywords:** bullectomy, bullous emphysema, COVID‐19 infection, giant bulla, vanishing lung syndrome

## Abstract

Bullous emphysema is a chronic disease characterized by bullae, or air spaces in the lungs. Giant bullae exceed one‐third of the hemithorax volume and are usually treated via bullectomy. We present the case of a 35‐year‐old man who presented to hospital with a history of COVID‐19 infection and seven days of chest pain and dyspnoea. A giant left upper lobe fluid‐filled bulla was identified on computed tomography. He was discharged with a course of antibiotics. A radiograph performed one month after presentation revealed an unchanged giant bulla. However, a chest radiograph and computed tomography nine months after initial presentation showed complete spontaneous resolution of the bulla. Bullectomy was deemed unnecessary. Cases of spontaneous bullae resolution, or autobullectomy, are rare. Our case implicates the role of infectious processes in autobullectomy. Serial imaging monitoring and delayed cardiothoracic assessment may be prudent to assess bullectomy necessity.

## Graphical abstract

We describe a rare case of autobullectomy in a 35‐year‐old man with a history of smoking and bullous emphysema over the course of 8 months. We believe that the findings of this case should be taken into consideration when contemplating surgery for patients with giant bullae.

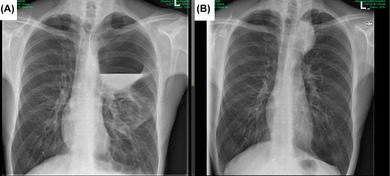

## INTRODUCTION

Emphysema is defined as ‘a condition of the lung characterized by abnormal, permanent enlargement of airspaces distal to the terminal bronchiole, accompanied by the destruction of their walls, and without obvious fibrosis’.^1^ It is often associated with chronic obstructive pulmonary disease. Bullous emphysema is a subtype of emphysema characterized by the formation of bullae, distended air spaces within the lung at least 1 cm in diameter. Giant bullae, also known as vanishing lung syndrome, are bullae that occupy at least 30% of the hemithorax.^2^

Patients with giant bullae can present with recurrent chest infections and worsening dyspnoea.^3^ The standard treatment for symptomatic giant bullae is bullectomy.^2^, ^4^ Spontaneous resolution, or autobullectomy, of giant bullae is rare but is described. We present a case of spontaneous resolution of a giant bulla in a young man with a history of COVID‐19 and superadded bacterial infection, followed by a literature review.

## CASE REPORT

A 35‐year‐old man was admitted to Charing Cross Hospital with a 7‐day history of slight left‐sided chest pain, shortness of breath, cough productive of yellow sputum, and fatigue. He described his chest pain as dull, aching, constant, worsening upon coughing and deep inspiration. He was afebrile and denied night sweats or weight loss. Ten days prior, he had tested positive for COVID‐19 and experienced fatigue and light‐headedness. He reported a 10‐pack‐year history of smoking cigarettes and occasional cannabis use. He had no prior medical history, regular medications, significant occupational exposure, or relevant family history.

His vital signs revealed mostly normal findings (temperature 36.5°C; blood pressure 143/74 mmHg; Glasgow Coma Scale 15; oxygen saturation 100% in room air) but increased respiratory rate (21 bpm) and heart rate (104 bpm). Chest examination revealed bronchial breath sounds in his left chest posteriorly. Remaining physical examinations were unremarkable.

Laboratory investigations revealed normal white cell count (10.6 × 10^9^/L) and Troponin T levels (<5 ng/L; normal range: <14 ng/L) but elevated C‐Reactive Protein (136 mg/L; normal range: 3–10 mg/L) and D Dimer levels (1900 ng/mL). Upon detection of the giant bulla, tests for Alpha‐1‐antitrypsin (A1AT) deficiency (1.7 g/L; normal range: 1.5–3.5 g/L) and HIV antibody were done, showing normal results. A sputum MC&S was not sent because the patient could not produce a sample.

**FIGURE 1:**
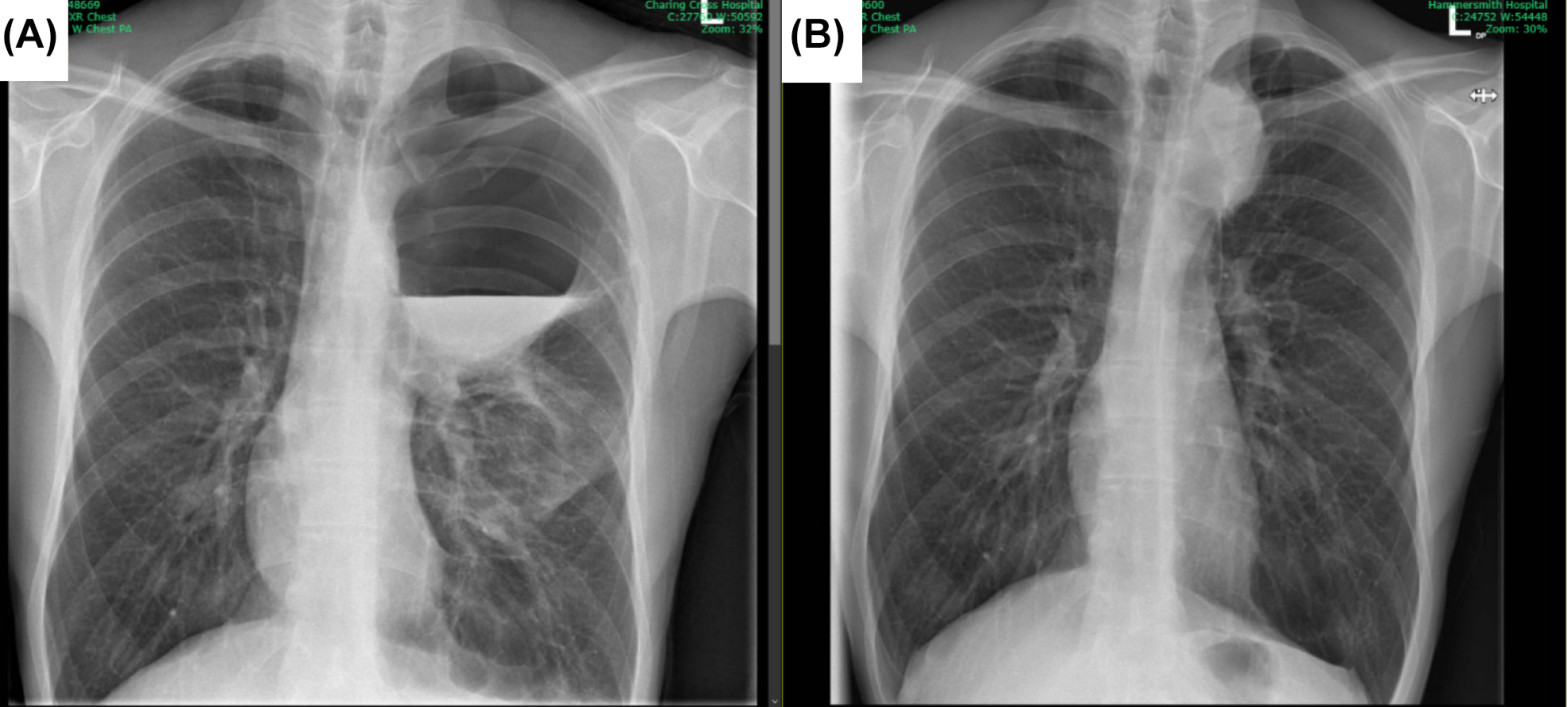
Side‐by‐side comparison of chest x‐rays on admission (A) and at 8 months (B) revealed the spontaneous resolution of the giant bulla in the left upper lobe.

**FIGURE 2:**
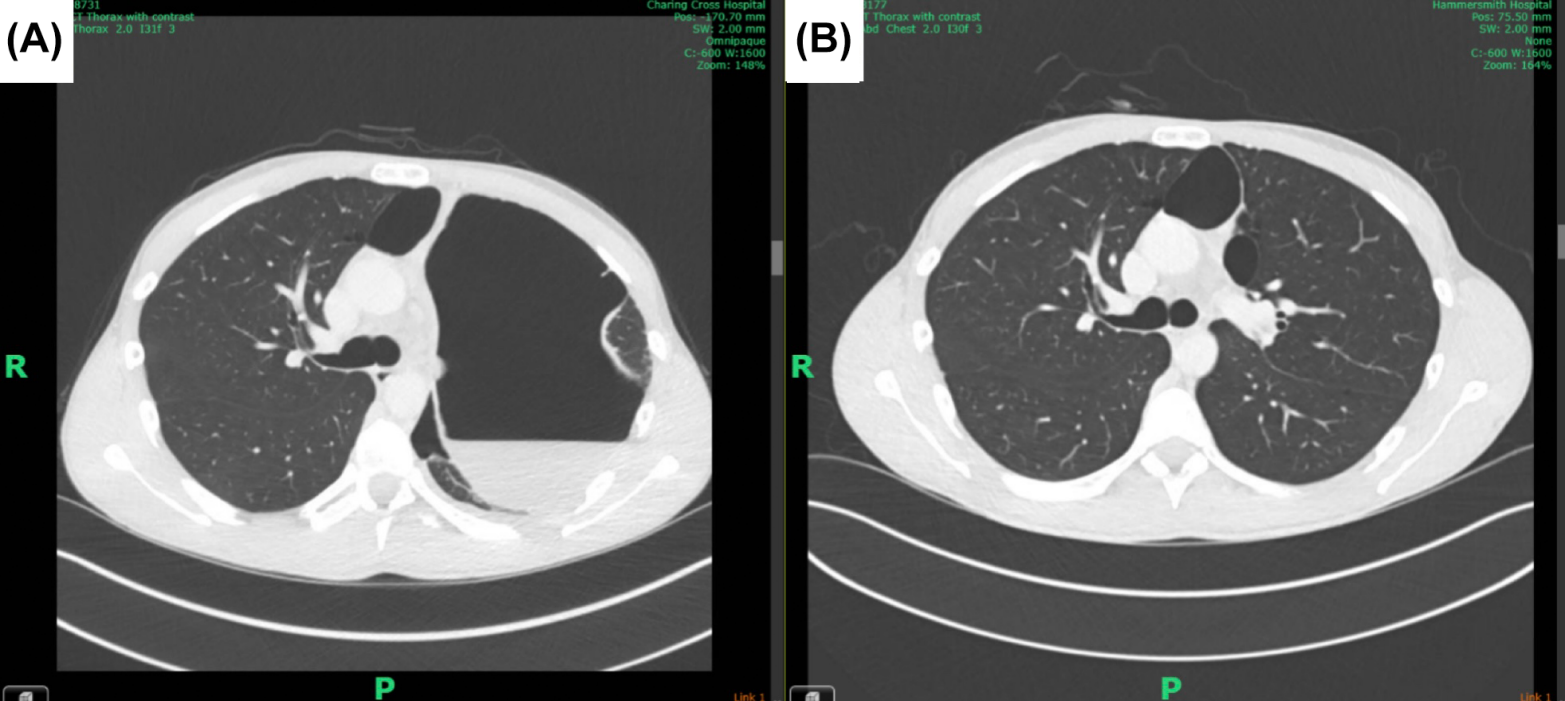
Side‐by‐side comparison of chest computed tomography on admission (A) and at 9 months (B) revealed the spontaneous resolution of the giant bulla in the left upper lobe.

Chest radiograph and computed tomography (CT) thorax (Figures 1A and 2A) taken on admission (there was no prior imaging) detected a giant air cavity, most likely a giant bulla, causing LUL collapse save for the inferior lingular segment, where there were patchy, ground‐glass opacities. A linear streak of confluent consolidation extended from the air cavity in the same region. The cavity's initial size was 120 × 146 mm (TR × CC) with a slightly thickened wall. Fluid levels in the bulla measured up to 4 cm in axial depth, likely being infected fluid. The giant bulla was surrounded by smaller bullae containing no fluid. Moderate‐sized bullae in the anterior right upper lobe (RUL) and right apex could also be detected. There was a small left pleural effusion but no significant lymphadenopathy.

The differential for chest radiograph appearances included hydropneumothorax. This was excluded on a CT thorax with contrast.

The patient was treated with a 10‐day course of oral co‐amoxiclav. A day later, his condition improved, showing normal vital signs (blood pressure 125/81 mmHg; pulse rate 76 bpm; respiratory rate 16 bpm). Repeated blood tests were unremarkable. He was discharged with a management plan to monitor the bulla and discuss bullectomy in the follow‐up review. A subsequent chest radiograph 1 month after initial presentation displayed clear pleural spaces and persistent air‐fluid level but no change in bulla structure. A pulmonary function test (PFT) undertaken at this time were as follows [FVC 7.45 L (125% predicted); FEV1 5.58 L (117% predicted); TLCO 12.08 mL·min^−1^·kPa^−1^ (92% predicted); KCO 1.31 mL·min^−1^·kPa^−1^ (84% predicted)]. Eight months later, the patient reported resolution of his chest pain and only mild exertional dyspnoea. A chest radiograph (Figure 1B) taken at cardiothoracic outpatient clinic review 9 months after initial presentation revealed that the bulla had shrunken markedly to 51 × 100 mm (TR × CC) with normal expansion of the LUL. A CT thorax (Figure 2B) with contrast confirmed complete spontaneous resolution of the giant bullae. The patient has since reported returning to daily life without issue.

## DISCUSSION

Bullous emphysema affects 5% of the global population and almost 12% of adults over 30.^5^ Aetiologies of bullous emphysema include smoking tobacco and cannabis,^6^, ^7^ HIV infection,^8^ COVID‐19 infection,^9^ lung surgery,^10^ and A1AT deficiency.^11^ Idiopathic bullous emphysema can also occur.^4^, ^12^ Pulmonary bullae are known to expand gradually. The exact mechanism is uncertain but could be due to the greater the elastic recoil of adjacent lung parenchyma relative to the bulla causing lung tissue to progressively retract outwards. This is supported by measurements from four preoperative patients showing that pressures within bullae were equivalent to pleural pressure irrespective of respiratory phases.^13^, ^14^ A more commonly‐accepted explanation is the check valve hypothesis, in which structures in the airway make gas entry into the bulla easier than exit, resulting in increasing positive‐end expiratory pressures and bulla expansion.^15^

Complete spontaneous resolution of giant bullae is rare. We searched the Medline and Embase databases using the searching terms ‘pulmonary emphysema’, ‘pulmonary bulla’, ‘vanishing lung syndrome’, ‘giant’, ‘regression’, ‘spontaneous’, and ‘autobullectomy’ from their respective start dates through May 1st 2024 and identified 14 other cases to date. Our patient was younger than the patients reported, who ranged from 45 to 74 years old. These patients were predominantly smokers or ex‐smokers. It is unclear why there are no documented cases of spontaneous resolution in females. The most probable explanation is the greater use of tobacco products among males in the past.^16^ Giant bullae were predominantly detected in the upper lung lobes. Reported resolution time, or the time from the onset of detection to detection of resolution, ranged from 2 weeks to 2 years. However, actual bulla resolution time is unclear. Resolution was often accompanied by symptom and PFT improvement. Some possible explanations attributed to resolution included bulla rupture into pneumothorax, benign nodule, and bronchodilator administration, with the most common explanation being bulla infection.^13^, ^16^, ^17^, ^18^, ^19^, ^20^, ^21^, ^22^, ^23^, ^24^, ^25^, ^26^ Eight of these cases involve peri‐emphysematous lung infection, or infection of the bullae, leading to the development of air fluid levels^27^ Infections are hypothesized to induce airway inflammation, which causes closure of communication between airway and bulla. Trapped gases can then be absorbed, leading to regression and bulla collapse.^16^, ^20^ Our patient's bulla resolution time was similar to other reported cases and was also associated with bulla infection, as evidenced by the detected air fluid level.

What makes this particular case of interest is the patient's recent infection with COVID‐19. We suspect that he may have had minor pre‐existing bullous emphysema as a result of smoking but that the giant bullae formed in response to infection. Airway inflammation is a common consequence of COVID‐19 infection, either directly or via secondary infection, and can lead to autobullectomy through the check valve mechanism.^24^, ^28^ Our patient presented similarly to a reported case of a 72‐year‐old man in Japan who showed partial regression in his emphysematous bulla 1 year after initial COVID‐19 infection and lung organizing pneumonia.^29^ Our findings emphasize the assumption that infectious processes play a part in bulla resolution (Table 1).

**TABLE 1:** Characteristics of patients in documented cases of complete giant bulla resolution.^13^, ^16^, ^17^, ^18^, ^19^, ^20^, ^21^, ^22^, ^23^, ^24^, ^25^, ^26^

Patient	Smoking status	Bulla location	Possible explanation for resolution	Reported resolution time	PFT outcome
62 Male^16^	Current	LUL	Bronchodilator and anti‐inflammatory	6 months	Improved
64 Male^11^	Former	LUL	Post‐infectious	2 years	Normal
55 Male^18^	Former	RUL	Post‐infectious	9 months	No change
74 Male^19^	Current	LUL	Post‐infectious	3 months	N/A
59 Male^20^	N/A	RUL	Post‐infectious	5 months	N/A
57 Male^20^	N/A	RUL	Post‐infectious	8 months	N/A
45 Male^20^	N/A	RUL	Post‐infectious	4 months	N/A
47 Male^21^	Former	LUL	Pneumothorax	6 months	Improved
54 Male^22^	Current	Right	Pneumothorax	3 years	Improved
70 Male^13^	Former	Right	Benign Nodule	2 years	Improved
57 Male^23^	N/A	LUL	N/A	1 month	N/A
67 Male^24^	Former	LLL	N/A	6 months	N/A
51 Male^25^	Former	RUL	Post‐infectious	1 year	Improved
63 Male^26^	Current	LUL	Post‐infectious	2 weeks	N/A

Abbreviations: LUL, left upper lobe; N/A, not available; RUL, right upper lobe.

Detection of giant bullae poses challenges towards its management. First, acutely detected giant bullae can mimic tension pneumothorax radiologically. Mistaking giant bullae for pneumothorax can lead to iatrogenic pneumothorax secondary to chest drain insertion. Careful review of historical imaging and a low threshold for obtaining cross sectional imaging can prevent this. The pleural line, which is concave to the lateral chest wall when associated with giant bulla but convex when associated with pneumothorax, distinguishes these conditions on medical imaging.^30^ Second, giant bullae are usually associated with respiratory symptoms or abnormal pulmonary function. Symptomatic patients are typically treated with bullectomy. However, bulla regression was often observed in situations where the patient has refused bullectomy,^18^, ^21^ was unfit for bullectomy,^1^, ^9^ or had to wait a prolonged period for bullectomy, like in the current case, giving the bulla a chance to regress before contemplating surgery. Consideration should be given to a delayed surgical approach, particularly in cases related to infection, to allow a window for spontaneous resolution. The optimal length of this period is currently unclear, but a duration of 6 months, which is the median duration of the aforementioned cases, is probably a reasonable time frame.

In conclusion, the possibility of autobullectomy in patients with giant bullae may warrant a delayed surgical approach. Due to the scarcity of literature, further investigation towards the underlying mechanisms, timeline, and contributing factors of autobullectomy is warranted.

## AUTHOR CONTRIBUTIONS

All persons listed as authors qualify for authorship as detailed in the author guidelines. Carl Reynolds and TT were involved in the clinical management of the patient. Carl Reynolds had the initial idea of writing this case report. Carl Reynolds, Ian Y. H. Chan, and KEJP discussed the case and to approach the first draft. Ian Y. H. Chan wrote the first draft. All authors reviewed and revised the manuscript and have agreed on the final version.

## FUNDING INFORMATION

No specific funding was provided for this case report. KEJP is funded by the National Heart and Lung Institute Clinical Lecturer scheme.

## CONFLICT OF INTEREST STATEMENT

None declared.

## ETHICS STATEMENT

The authors declare that appropriate written informed consent was obtained for the publication of this manuscript and accompanying images.

## Data Availability

The data that support the findings of this study are available on request from the corresponding author. The data are not publicly available due to privacy or ethical restrictions.
